# Preparation and Characterization of Vancomycin Hydrochloride-Loaded Mesoporous Silica Composite Hydrogels

**DOI:** 10.3389/fbioe.2022.826971

**Published:** 2022-02-08

**Authors:** Ming Sun, Lidi Cheng, Zexian Xu, Liqiang Chen, Yanshan Liu, Yaoxiang Xu, Dongyang Zhou, Xiuxiu Zhang, Qihui Zhou, Jian Sun

**Affiliations:** ^1^ The Affiliated Hospital of Qingdao University, Qingdao, China; ^2^ School of Stomatology of Qingdao University, Qingdao, China; ^3^ Dental Digital Medicine & 3D Printing Engineering Laboratory of Qingdao, Qingdao, China; ^4^ Institute for Translational Medicine, The Affiliated Hospital of Qingdao University, Qingdao, China; ^5^ Shandong Provincial Key Laboratory of Digital Medicine and Computer‐Assisted Surgery, Qingdao, China

**Keywords:** biomaterials, hydrogel, drug delivery, biocompatibility, antibacterial

## Abstract

This study aims to explore the feasibility of the novel temperature-sensitive hydrogel-based dual sustained-release system (Van/SBA-15/CS-GP-SA) in the repair and treatment of infectious jaw defects. Van/SBA-15 was prepared using the mesoporous silica (SBA-15) as a carrier for vancomycin hydrochloride (Van), and Van/SBA-15 was characterized by scanning electron microscopy (SEM), transmission electron microscopy (TEM), energy dispersive spectrometry (EDS), X-ray photoelectron spectroscopy (XPS), Fourier transform infrared (FTIR), Brunauer–Emmett–Teller (BET), and Barrett–Joyner–Halenda (BJH). The characterization results confirm that Van is loaded in SBA-15 successfully. Van/SBA-15/CS-GP-SA is constructed by encapsulating Van/SBA-15 in chitosan–sodium glycerophosphate–sodium alginate hydrogel (CS-GP-SA). The microstructures, sustained-release ability, biocompatibility, and antibacterial properties of Van/SBA-15/CS-GP-SA were systematically studied. Van/SBA-15/CS-GP-SA is found to have promising sustained-release ability, outstanding biocompatibility, and excellent antibacterial properties. This study provides new ideas for the management of infectious jaw defects.

## 1 Introduction

The number of cases of jaw defects caused by accidents, trauma, cancer, congenital malformations, and other related diseases is increasing, with infected bone defects caused by wound contamination being a special type of bone defect (Darwich et al., 2021; Khorasani et al., 2021; Wang et al., 2021b). Until now, repairing infected jaw defects has remained a challenge due to its high recurrence rate for oral and maxillofacial surgeons (Zhang et al., 2019). To our knowledge, infection is due to the local presence of pathogenic bacteria in the lesion, and once an infected jaw defect occurs, it may require multiple surgical debridements and long-term systemic antibiotic therapy, which may lead to the development of secondary injury, adverse antibiotic reactions, or lifelong functional impairment, and increase the financial burden on the health-care system (Vestby et al., 2020; Wang et al., 2020). Therefore, antimicrobial drug carriers, a topical strategy, have become an adjunct to the prevention and treatment of infections in jaw defects (Alazzawi et al., 2021; Han et al., 2021). Topical administration at or near the site of infection diminishes the toxic effects of antibacterial agents, reduces the required dose of antibiotics, and enhances the bioavailability and safety of the drug (Wychowański et al., 2021; Zheng et al., 2021).

At present, vancomycin hydrochloride (Van), a glycopeptide antibiotic, has been widely used to treat infectious jaw defects (Ahadi et al., 2019). However, due to the short half-life of Van, patients need to be injected with it repeatedly, which may cause adverse effects in the organism (Pecci et al., 2020). SBA-15, a type of ordered mesoporous silica material with a two-dimensional hexagonal pore structure, is a promising carrier for the controlled release of therapeutic agents (Alkafajy and Albayati, 2020; Esperanza Adrover et al., 2020; Seljak et al., 2020; Sun et al., 2019). Large pore volume, size, and high surface area of SBA-15 contribute to an improved drug loading rate (Bavnhøj et al., 2019; Alkafajy and Albayati, 2020). Its mesoporous structure helps improve drug stability and prolong drug release time (Moritz and Marek, 2012; Shen et al., 2020). Hydrogels are also widely used as carriers in drug release because of their ability to modulate the rate of drug release effectively (Zhao et al., 2020). Chitosan (CS) has antibacterial properties, excellent cytoadhesive properties, biocompatibility, and stability (Abinaya et al., 2019; Li et al., 2020; Cui et al., 2021). The positive charge on CS can inhibit the growth and reproduction of some bacteria and viruses, which is beneficial to reduce the infection (Abid et al., 2019; Hao et al., 2021; Yin et al., 2021). The three-dimensional network structure of chitosan temperature-sensitive hydrogels can provide a good microenvironment for cell proliferation, migration, and differentiation and can guide the long entry of host cells (Zhao et al., 2020; Kang et al., 2021; Sordi et al., 2021; Yang et al., 2021). Sodium alginate (SA) is a natural polysaccharide that is a by-product of iodine and mannitol extraction from the brown algae kelp or sargassum, which has promising biocompatible and biodegradable (Mujtaba and Alotaibi, 2020). Studies have shown that CS forms a cross-linked structure with SA through electrostatic interaction and hydrogen bonding, which strengthens the linkage between the molecular chains of sodium alginate, thus enhancing the mechanical properties and release control ability of the hydrogel (Kiti and Suwantong, 2020; Li et al., 2020; Kayan and Kayan, 2021).

To explore the feasibility of the dual sustained-release hydrogel system in the repair and treatment of infectious jaw defects, Van/SBA-15/CS-GP-SA was prepared and characterized.

## 2 Materials and Methods

### 2.1 Materials

Van and SA were purchased from Dalian Meilun Biotechnology Co., Ltd. (Dalian, China). SBA-15 was provided by Nanjing XFNANO Materials Tech Co., Ltd. CS (deacetylation degree ≥95%) and acetic acid (purity ≥99.8%) were obtained from Shanghai Macklin Biochemical Co., Ltd. (Shanghai, China).

### 2.2 Preparation of Van/SBA-15

Hundred milligrams of SBA-15 was dissolved in 10 ml of double-distilled water and then sonicated to obtain SBA-15 suspension. Van powder (20 mg) was added to the SBA-15 suspension, and the mixture was stirred for 8 h at room temperature. Further 2 h of vacuum treatment was then carried out in a vacuum oven to facilitate the encapsulation of the Van in SBA-15. Subsequently, the mixture was centrifuged (8,000 rpm, 10 min) and washed twice with double-distilled water. The resultant Van/SBA-15 was stored at 4°C. Meanwhile, to confirm the drug encapsulation efficiency, the supernatant and wash solution were collected and analyzed at a wavelength of 280 ± 2 nm using an ultraviolet–visible (UV) spectrophotometer (Aucybest, China). The encapsulation efficiency of Van/SBA-15 was 36.8%.

### 2.3 Characterization of Van/SBA-15

SBA-15 and Van/SBA-15 were characterized by SEM (JSM-7001F, Japan) and TEM (Jeol/JEM 2100, USA). For SEM observation, samples were sputter-coated with gold to increase electronic conductivity. And TEM images were taken with a JEOL JEM 2100 electron microscope at an accelerating voltage of 200 kV. The elemental profiles were analyzed by EDS (IXRF550i, USA).

The element compositions were detected by XPS (ESCALAB 250XI, USA). FTIR spectra of SBA-15 and Van/SBA-15 were measured using the KBr pellet method on a Nicolet 6700 spectrometer (Thermo, USA) from 4,000 to 400 cm^−1^. The BET and BJH analyses were employed to determine the pore size distribution, pore diameter, and specific surface area, through N_2_ adsorption–desorption isotherms (V-Sorb 2800P analyzer, Gold APP, China).

### 2.4 Preparation and Characterization of Van/SBA-15/CS-GP-SA

Sodium glycerophosphate (GP, 600 mg) was dissolved in 1 ml of double-distilled water. After that, 40 mg of SA was added to the solution. The resultant GP-SA solution was refrigerated at 4°C. Chitosan (250 mg) was dissolved in 10 ml of 0.1 mol/L acetic acid and then stirred for 2 h. GP-SA and a certain amount of Van/SBA-15 were added to the pre-prepared chitosan solution and then stirred for 30 min. The obtained sol was poured into a mold soaked in a 37°C water bath to produce Van/SBA-15/CS-GP-SA gel (Laurano et al., 2020). The morphology of CS-GP-SA, Van/CS-GP-SA, and Van/SBA-15/CS-GP-SA were observed by SEM and TEM.

### 2.5 Investigation of *In Vitro* Drug Release

*In vitro* drug release was analyzed by using the dialysis method (Ma and Shi, 2019). Van standard solution was diluted to different concentrations (200–1,000 μg/ml), and the absorbance at 280 ± 2 nm of the solutions was measured. The standard curve was constructed by plotting concentration against absorbance. Each Van/SBA-15, Van/CS-GP-SA, and Van/SBA-15/CS-GP-SA was weighed to 200 mg and then placed in a dialysis bag. The dialysis bag was placed in a beaker containing a release medium (10 ml of PBS, pH = 7.4), sealed, and then shaken at a constant speed at 37°C. The release solution (3 ml) was collected at different time points and stored until further tests, while equal amounts of PBS were supplemented to the solution. UV absorbance at 280 ± 2 nm was monitored. The concentrations were calculated based on the standard curves, and the cumulative release of Van at different time points was calculated.

### 2.6 Analysis of *In Vitro* Cytocompatibility

The samples used in this experiment were divided into four groups: control, CS-GP-SA, Van/CS-GP-SA, and Van/SBA-15/CS-GP-SA. The hydrogels were sterilized under ultraviolet light for 2 h immediately after manufacture and then immersed in a cell culture medium at a surface area of 1 ml/cm^2^ of hydrogel for 24 h at 37°C to extract the compounds. The MT3C3-E1 cells were seeded in 96-well plates at 5 × 10^3^ cells/well. Five replicate samples in each group were prepared. The plates were incubated at 37°C in an incubator saturated with 5% CO_2_ for 24 h (Ji et al., 2021). After the cells adhered to the bottom of the well, the supernatant was discarded. Then the extract (200 µl) was added to the CS-GP-SA, Van/CS-GP-SA, and Van/SBA-15/CS-GP-SA group, and the cell culture medium at the same volume was added to the control group. After continuously incubated for 1, 3, and 5 d, the numbers of viable cells were counted using the Cell Counting Kit-8 (CCK-8) (Sigma). CCK-8 solution (20 μl) was added to each well, and the absorbance at 450 nm was measured after 1 h. For the quantification of cell number, 4,6-diamidino-2-phenylindole (DAPI) was used to stain the cell nuclei (Pierozan and Karlsson, 2019).

### 2.7 Analysis of *In Vitro* Antibacterial Activity

The hydrogel was sterilized under ultraviolet light for 2 h before use (Op't Veld et al., 2020). Then 200 μl suspension of *Escherichia coli* (*E. coli*) and *Staphylococcus aureus* (*S. aureus*) were spread evenly on a Luria–Bertani (LB) agar plate and then incubated 37°C for 1 h. CS-GP-SA, Van/CS-GP-SA, and Van/SBA-15/CS-GP-SA with a diameter of 1 cm were placed on the LB agar plate containing the bacteria and then incubated at 37°C for another 24 h in a bacterial incubator; Van/CS-GP-SA and Van/SBA-15/CS-GP-SA contain an equal amount of vancomycin. After that, the diameters of the inhibition zones were measured. The data were the average of the three replicate samples.

### 2.8 Statistical Analysis

Statistical analysis was conducted using SPSS version 26.0. One-way ANOVA and Tukey’s multiple comparison method were used to determine the significant differences between groups. The differences with *p* < 0.05 were considered statistically significant.

## 3 Results and Discussion

### 3.1 Characteristics of Van/SBA-15

**FIGURE 1:**
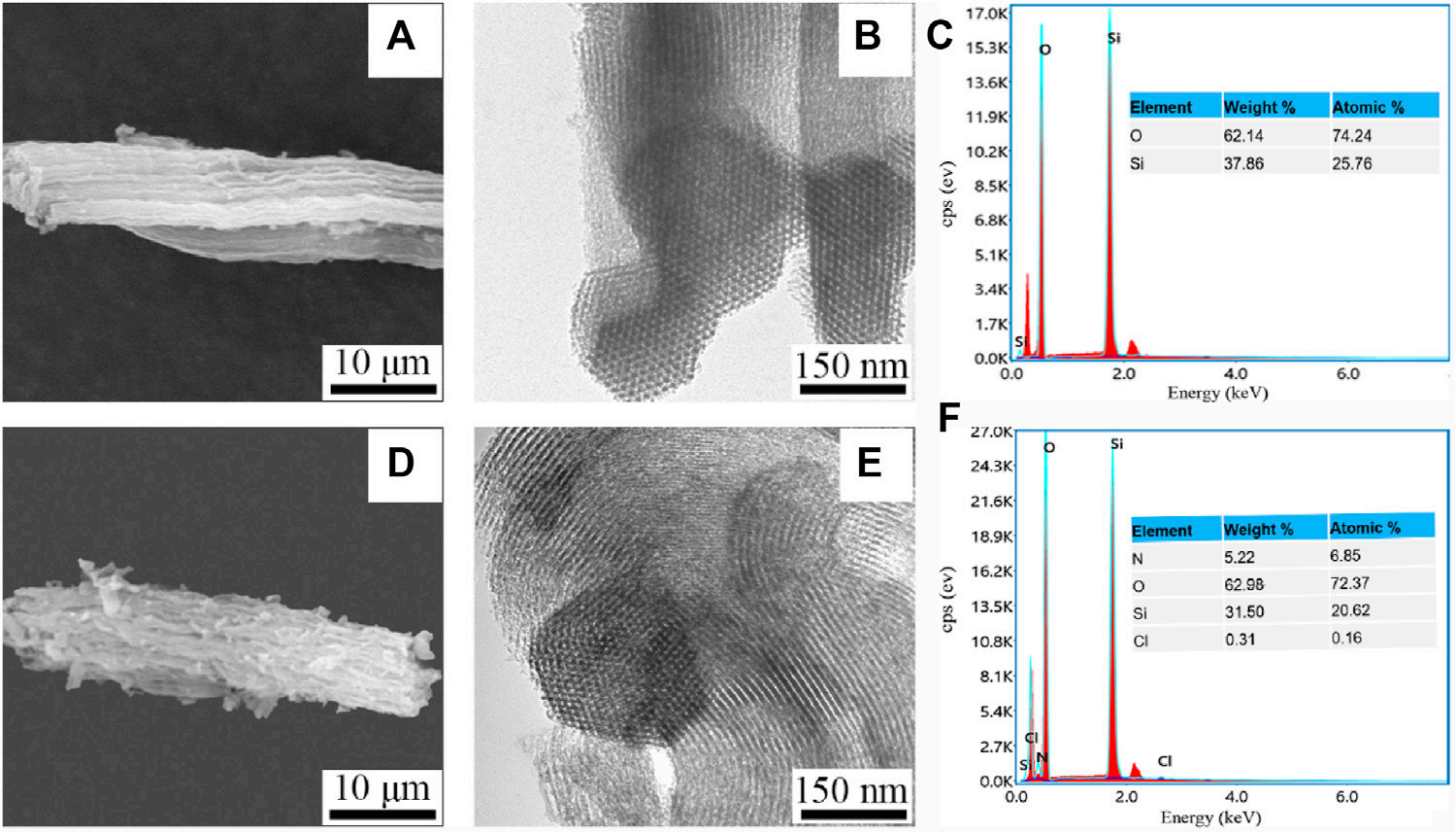
SEM images of SBA-15 **(A)** and Van/SBA-15 **(D)**, TEM images of SBA-15 **(B)** and Van/SBA-15 **(E)**, EDS analysis of SBA-15 **(C)**, and Van/SBA-15 **(F)**.

The SEM image showed that SBA-15 microscopically consisted of many short cylindrical rod-shaped particles with relatively uniform sizes, which is the typical morphological feature of SBA-15 molecular sieves. The surface morphologic image of Van/SBA-15 clearly showed that Van was adsorbed on the surface of SBA-15, which is an indication that the surface of SBA-15 has the capacity to adsorb drugs (Figures 1A,D). The TEM image showed that SBA-15 had a two-dimensional hexagonal structure. The pores of Van/SBA-15 became blurred, suggesting that Van was successfully loaded into SBA-15 (Figures 1B,E). The EDS spectrum showed that SBA-15 contained O and Si, and Van/SBA-15 contained not only O and Si but also N and Cl (Figures 1C,F). Van/SBA-15 contained two elements, N and Cl, which are not present in SBA-15, and these two elements are formally Van’s elements. These EDS results further verify the TEM result.

**FIGURE 2:**
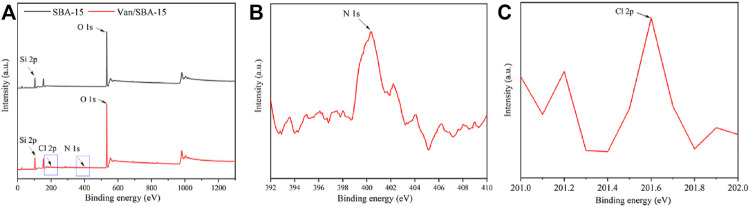
XPS images of SBA-15 and Van/SBA-15 **(A)**, XPS image of the N1s peak of Van/SBA-15 **(B)**, and XPS image of the Cl2p peak of Van/SBA-15 **(C)**.

Equally, the XPS results showed that Van/SBA-15 powder exhibited the N1s peak at 400.3 eV and Cl2p peak at 201.6 eV, while N1s and Cl2p peak did not appear in SBA-15 powder (Figure 2). The XPS results prove from the side that Van is successfully loaded in SBA-15.

**FIGURE 3:**
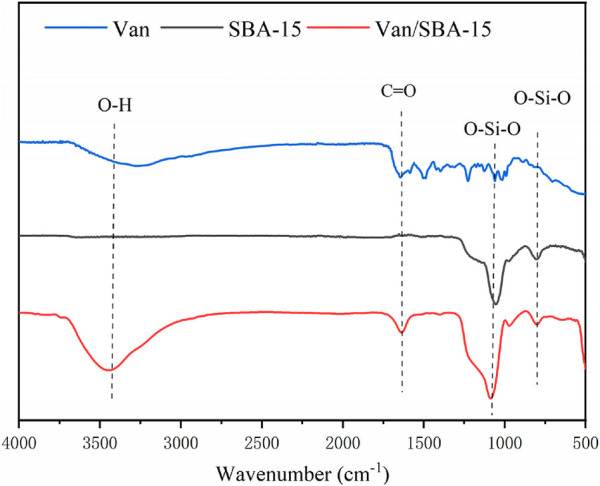
FTIR spectra of Van, SBA-15, and Van/SBA-15.

The FTIR spectrum of Van showed peaks at 3,440 cm^−1^ and 1,635 cm^−1^, which corresponded to the -OH stretching and the C=O vibration, respectively. These peaks are the characteristic peaks of Van. Furthermore, characteristic peaks corresponding to the stretching and bending of Si-O-Si in SBA-15 appeared at 1,090 cm^−1^ and 795 cm^−1^ (Kayan, 2016). The FTIR spectrum of Van/SBA-15 showed all the characteristic peaks of Van and SBA-15, confirming that Van was successfully encapsulated in SBA-15 (Figure 3).

**FIGURE 4:**
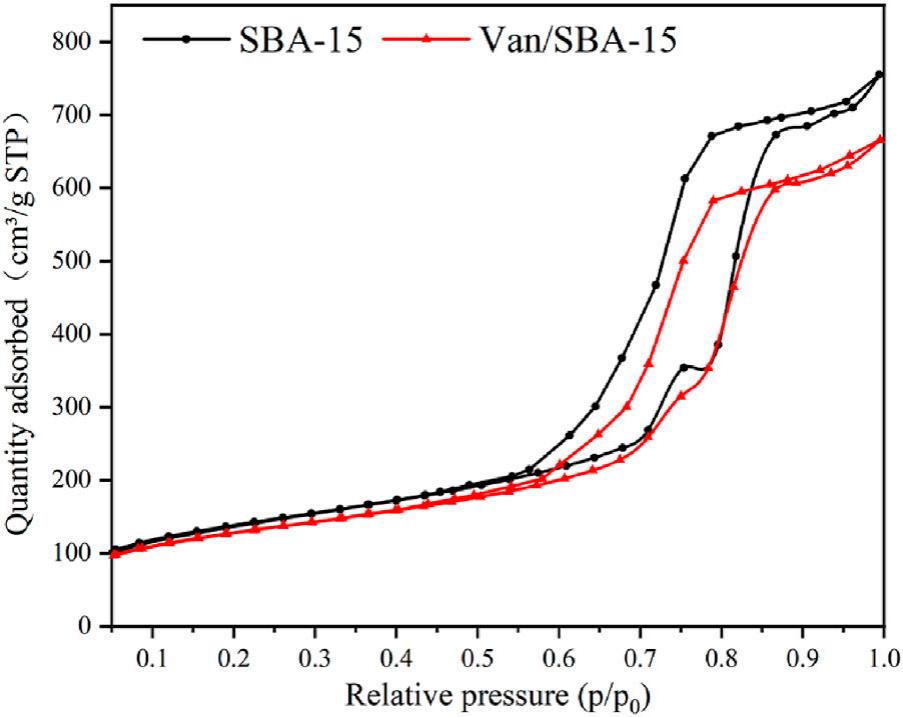
N_2_ adsorption–desorption isotherms of SBA-15 and Van/SBA-15.

The N_2_ adsorption–desorption isotherms before and after drug loading conformed to the type IV adsorption curve, which is the typical adsorption isotherms of mesoporous materials. The N_2_ adsorption after drug loading was significantly lower than that before drug loading, and both exhibited adsorption isotherm type IV and contained hysteresis loop (Figure 4). From the data given in Table 1, the loading of drug into the mesoporous pores of the material had no effect on the skeletal structure of SBA-15 but caused the reduction of pore size, pore volume, and specific surface area. This indicates that Van successfully entered into the pore channels of SBA-15 but not completely filled the pore channels.

**TABLE 1:** Pore parameters of SBA-15 and Van/SBA-15.

Sample	BJH adsorption average pore diameter	BJH adsorption cumulative volume of pores	BET surface area
SBA-15	10.6 m	1.1 cm^3^/g	465.4 m^2^/g
Van/SBA-15	10.2 nm	0.9 cm^3^/g	445.6 m^2^/g

### 3.2 Characteristics of Van/SBA-15/CS-GP-SA

**FIGURE 5:**
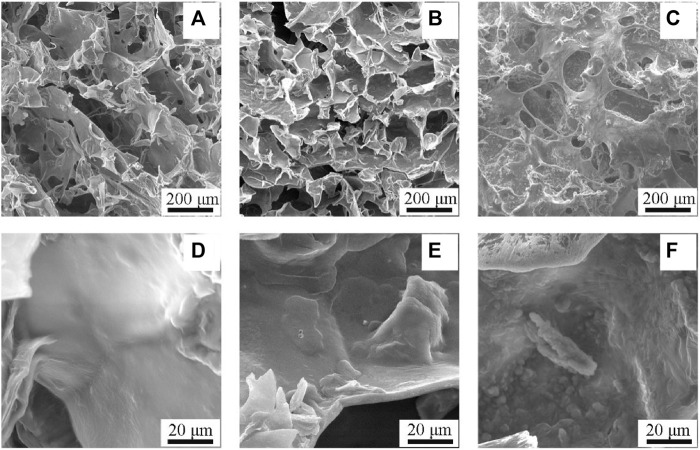
SEM images of CS-GP-SA **(A, D)**, Van/CS-GP-SA **(B, E)**, and Van/SBA-15/CS-GP-SA **(C, F)**.

The SEM image showed that samples in all the 3 groups had distinct porous structures. The pore walls of CS-GP-SA were smooth. When Van was added, the pore walls of the prepared Van/CS-GP-SA became rough, and the surface showed a protruding granularity. With the addition of Van/SBA-15, the pore walls of the prepared Van/SBA-15/CS-GP-SA were rougher, and the surface presented a more pronounced granularity. Similarly, we found that the addition of Van and Van/SBA-15 affected the pore size of the scaffolds. The pore size of the manufactured Van/SBA-15/CS-GP-SA was larger than the other two groups (Figure 5). This indicates that the addition of Van/SBA-15 to CS-GP-SA has a significant influence on the surface structure (Jaidev and Chatterjee, 2019; Ouyang et al., 2019; Metwally et al., 2020; Wychowański et al., 2021).

### 3.3 *In Vitro* Drug Release

**FIGURE 6:**
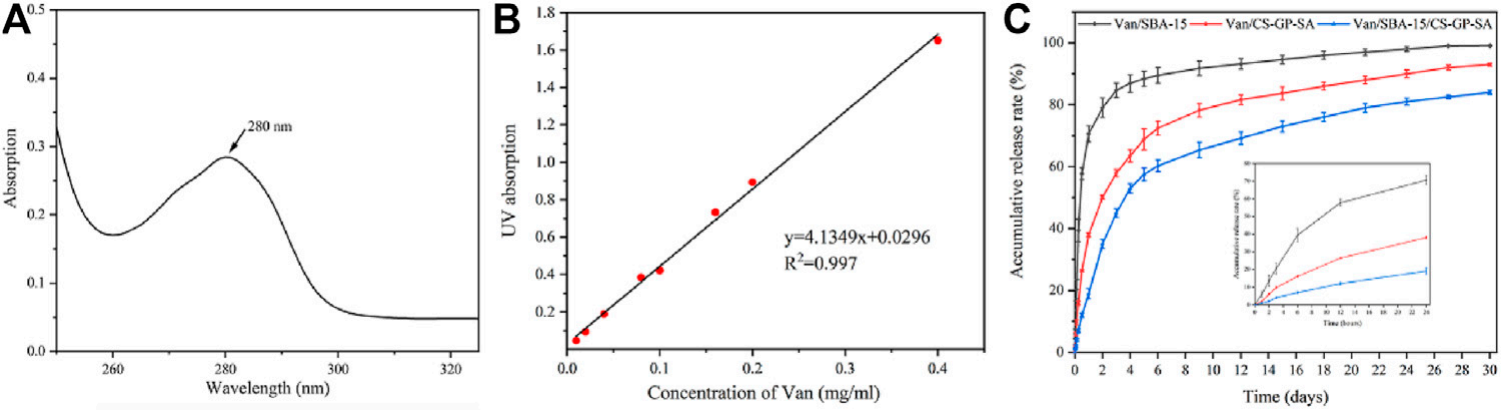
Ultraviolet absorption of Van solution **(A)**, the standard curve of Van solution **(B)**, the cumulative release profiles of Van from Van/SBA-15, Van/CS-GP-SA, and Van/SBA-15/CS-GP-SA **(C)**.

The controlled release curves of Van/SBA-15, Van/CS-GP-SA, and Van/SBA-15/CS-GP-SA showed that Van initiated burst release within the first 4 d and then sustained release thereafter. However, the slower release was observed in Van/CS-GP-SA and Van/SBA-15/CS-GP-SA. The cumulative release of Van from Van/SBA-15, Van/CS-GP-SA, and Van/SBA-15/CS-GP-SA on day 15 was (94.6 ± 1.37)%, (83.7 ± 2.10)%, and (73 ± 1.76)%, respectively, and that on day 30 was (99.1 ± 0.13)%, (93 ± 0.47)%, and (84 ± 0.64)%, respectively (Figure 6). These results suggest that the sustained-release of Van/SBA-15/CS-GP-SA was much better than those in other samples, which is probably due to the drug combined with nanoparticles by hydrogen bonding, ionic interaction, and physical absorption (Wang et al., 2021a).

### 3.4 *In Vitro* Cytocompatibility

**FIGURE 7:**
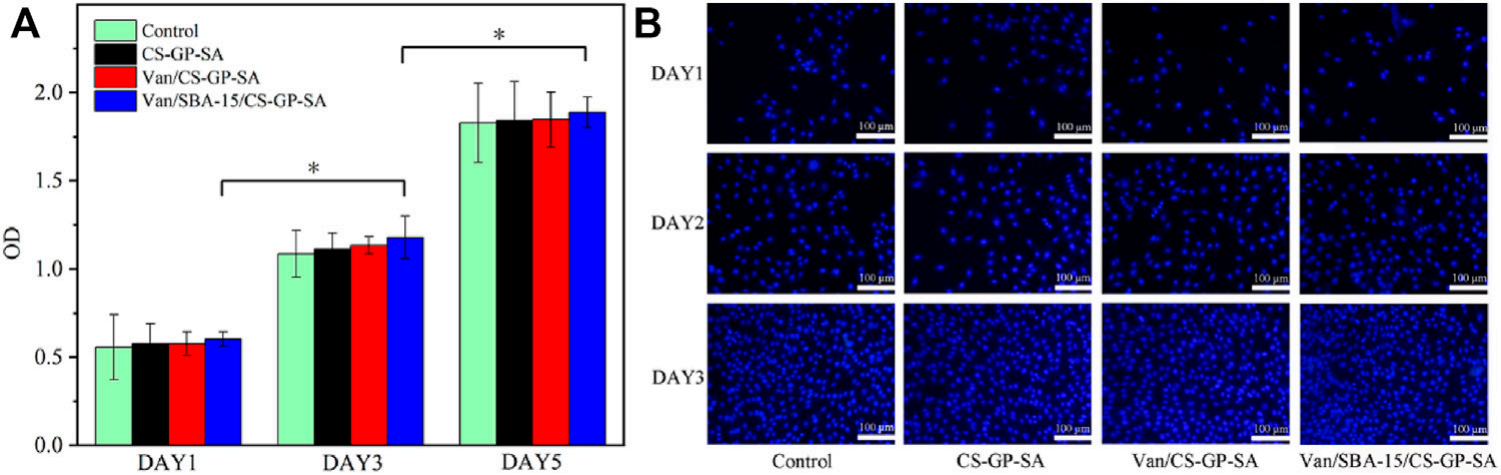
CCK-8 data **(A)** and DAPI staining **(B)** of control, CS-GP-SA, Van/CS-GP-SA, and Van/SBA-15/CS-GP-SA.

The CCK-8 data of all the four groups observed on 1, 3, and 5 d were not significantly different (*p* > 0.05). In addition, the cell proliferation indicated by the optical density (OD) values in all four groups increased with time (*p* < 0.05) (Figure 7A). The result was again confirmed with the nuclear staining method using DAPI for 1, 3, and 5 d. Figure 7B shows that all groups achieved a gradual increase in cell numbers with increasing incubation time, and there was no significant difference between the groups. The results indicate that the prepared composite hydrogels have good cytocompatibility and allow cell spreading and proliferation. The above results confirm that Van/SBA-15/CS-GP-SA has high cytocompatibility.

### 3.5 *In Vitro* Antibacterial Effect

**FIGURE 8:**
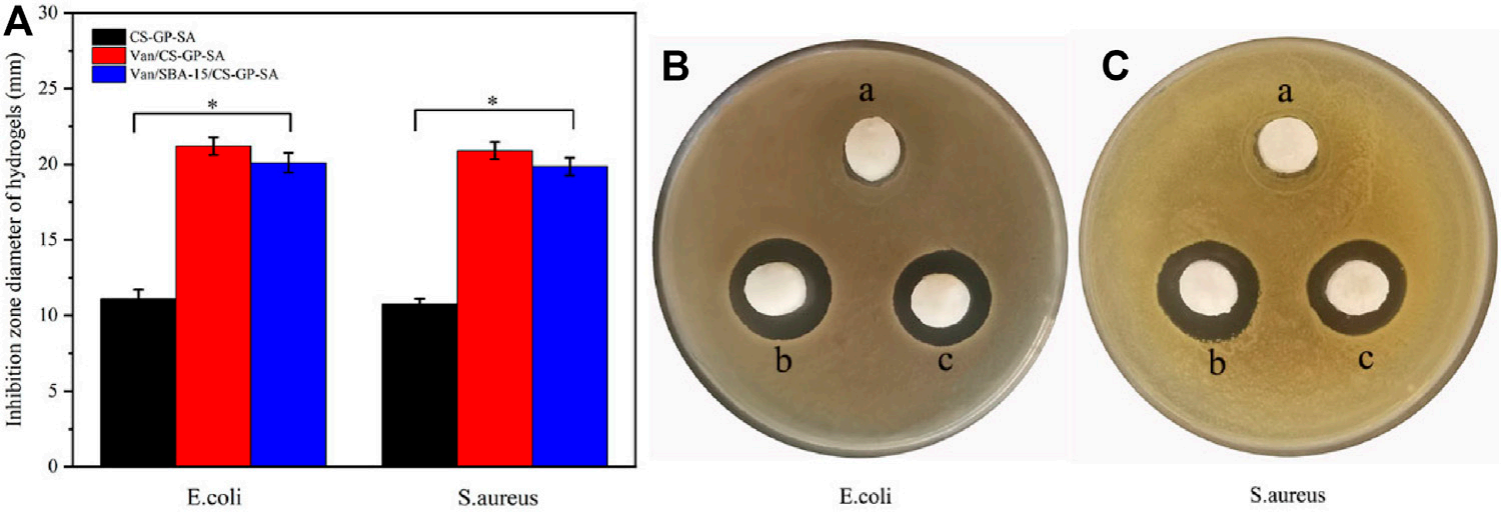
Quantitative evaluation of *in vitro* antibacterial effect. **(A)** Antibacterial test results for *E. coli* **(B)** and *S. aureus* **(C)** of CS-GP-SA (a), Van/CS-GP-SA (b), and Van/SBA-15/CS-GP-SA (c).

The bacteriostatic circle was more obvious in Van/SBA-15/CS-GP-SA and Van/CS-GP-SA than that in CS-GP-SA (*p* < 0.05). A less pronounced antimicrobial cyclic appeared around CS-GP-SA, and this may be caused by the antibacterial activity of CS (Zhu et al., 2021) (Figures 8B,C). These results indicate that Van/SBA-15/CS-GP-SA has excellent antibacterial properties.

However, as can be seen in the bar chart, Van/CS-GP-SA was slightly better than Van/SBA-15/CS-GP-SA, and this may be due to the slow release of SBA-15 (Figure 8A). The aformentioned finding further confirms that Van/SBA-15/CS-GP-SA has a sustained-release ability.

## 4 Conclusion

In this study, a novel temperature-sensitive hydrogel-based dual sustained-release system (Van/SBA-15/CS-GP-SA) was successfully prepared by Van-loaded SBA-15 and chitosan–glycerophosphate–alginate–sodium hydrogel, which have promising sustained-release ability, excellent biocompatibility, and antibacterial property. This study provides ideas for exploring new strategies for the management of infectious jaw defects.

## Data Availability

The raw data supporting the conclusion of this article will be made available by the authors, without undue reservation.
